# Molecular Responses of Lactobacilli to Plant Phenolic Compounds: A Comparative Review of the Mechanisms Involved

**DOI:** 10.3390/antiox11010018

**Published:** 2021-12-22

**Authors:** Félix López de Felipe, Blanca de las Rivas, Rosario Muñoz

**Affiliations:** Laboratorio de Biotecnología Bacteriana, Instituto de Ciencia y Tecnología de los Alimentos y Nutrición (ICTAN-CSIC), 28040 Madrid, Spain; blanca.r@csic.es (B.d.l.R.); rmunoz@ictan.csic.es (R.M.)

**Keywords:** plant phenolic compounds, molecular responses, system-based approaches, *Lactobacillus*

## Abstract

Lactobacilli are well-studied bacteria that can undergo oxidative selective pressures by plant phenolic compounds (PPCs) in plants, during some food fermentations or in the gastrointestinal tract of animals via dietary inputs. Lactobacilli are known to be more tolerant to PPCs than other bacterial groups and, therefore, must have mechanisms to cope with the effects of these metabolites. In this review, we intend to present what is currently known about the basics beyond the responses of *Lactobacillus* spp. to individual PPCs. We review the molecular mechanisms that are engaged in the PPC-modulated responses studied to date in these bacteria that have been mainly characterized by system-based strategies, and we discuss their differences and similarities. A wide variety of mechanisms are induced to increase the oxidative stress response highlighting the antimicrobial nature of PPCs. However other uncovered mechanisms that are involved in the response to these compounds are reviewed, including the capacity of PPCs to modulate the expression of molecular functions used by lactobacilli to adapt to host environments. This shows that these phytochemicals can act as more than just antimicrobial agents in the dual interaction with lactobacilli.

## 1. Introduction

Plant phenolic compounds (PPCs) are a class of phytochemicals with high diverse structural complexity. According to animal and in vitro studies that evidenced antioxidant, radical-scavenging, and antimutagenic properties, long-term consumption of different PPCs has been correlated with chronic disease prevention [1,2]. An important role attributed to PPCs is defense functionality against microbiological threats. Antimicrobials usually feature the overproduction of reactive oxygen species (ROS) to exert their activity [3]; therefore, oxidative stress can be at the basis of the antimicrobial action of PPCs. Owing to their antimicrobial properties PPCs may exert broad effects on microorganisms that can result in rapid shifts and long-term modification in plant and animal microbiomes. In this regard, several studies have shown that supplementation with food substrates rich in PPCs variates the structure of the gut microbiota, commonly resulting in alteration of the Firmicutes to Bacteroidetes ratio and leading to *Lactobacillus* spp. as one of the predominant bacterial populations [4,5,6,7,8]. In addition, supplementation with individual PPCs can also exert growth-promoting effects on *Lactobacillus* spp., which has been observed for flavonols [9,10], flavanols [11,12], hydroxybenzoic acids [13], flavonones, flavones and isoflavones [14], anthocyanins [15], hydrolyzable tannins [16], and stilbenes [17].

Improved knowledge on the molecular bases that govern the reciprocal polyphenol-microbiota interactions is important in two ways. On one hand, this knowledge is crucial to understand how PPCs shape host–microbial communities, on which host fitness partly depends. On the other hand, this knowledge is necessary to decipher the biochemical pathways that bacteria, in the context of symbiosis with their hosts, have evolved to metabolize PPCs and provide these compounds in their bioavailable and bioactive forms [2,18].

Although PPCs can exert broad effects on bacteria and shape host–microbial communities impacting host fitness, many knowledge gaps still remain on how bacterial physiology and functionality are influenced upon exposure to PPCs. In this regard, molecular techniques may help to increase our understanding of the mechanisms underlying the bacterial responses to PPCs. Approaches considering the use of individual PPCs appear to be more suitable than the application of plant substrates containing a mixture of PPCs which may limit a mechanistic understanding of the microbe–PPC interaction. *Lactobacillus* spp. are more tolerant to PPCs than other bacterial groups and are part of the microbiomes of humans [19], animals [20], and plants [21,22]; therefore, they are suitable to study the molecular interaction with PPCs.

This review summarizes the progress in our understanding of the molecular mechanisms underlying the responses of *Lactobacillus* spp. to individual PPCs. The differences and similarities between these responses are described and discussed.

## 2. Comparative Transcriptomic Responses of *L. plantarum* WCFS1 to Different PPCs

**Figure 1:**
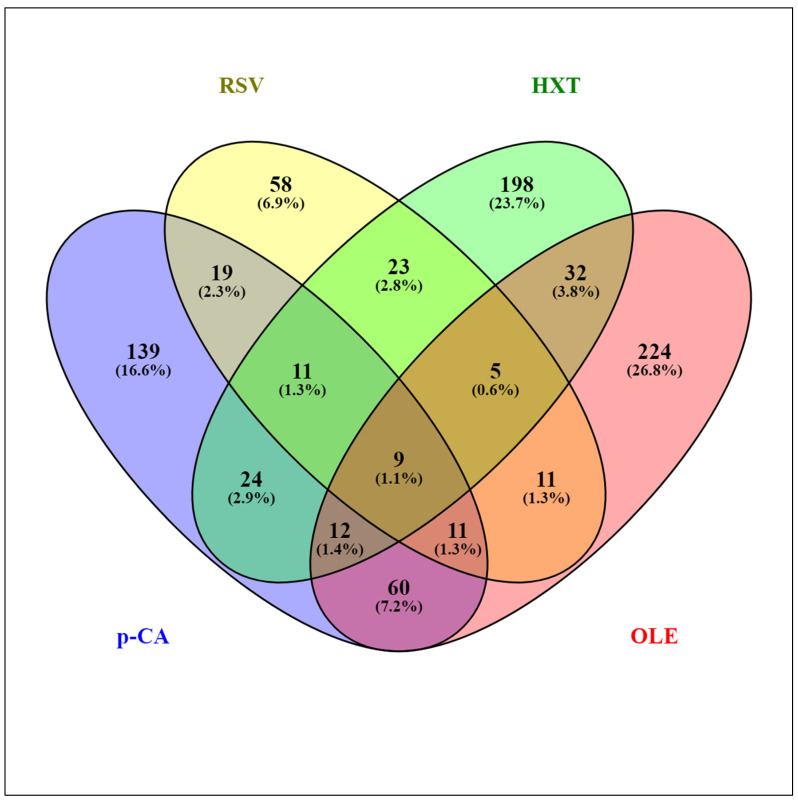
Venn diagram assembled from *Lactiplantibacillus plantarum* WCFS1 genes differentially expressed in response to various plant phenolic compounds: *p*-coumaric acid (*p*-CA), resveratrol (RSV), hydroxytyrosol (HXT), and oleuropein (OLE).

Several studies have addressed the transcriptomic responses of *Lactobacillus* spp. to individual PPCs [23,24,25,26,27,28,29]. The transcriptomes of the model strain *Lactiplantibacillus plantarum* WCFS1 exposed to different representative PPCs, including a hydroxycinnamic acid (*p*-coumaric acid (*p*-CA)) [25], a hydroxybenzoic acid (gallic acid (GA)) [26], a stilbene (resveratrol (RSV)) [27], a secoiridoid (oleuropein (OLE)) [28], and a phenylethanoid (hydroxytyrosol (HXT)) [29] have been revealed using DNA microarrays. The standardized experimental setup, applying stresses for 10 min to the same strain, permitted a reliable comparison among the different responses to these PPCs. The presence of PPCs affected hundreds of genes, and the largest transcriptome variations were observed upon exposure to OLE (358 genes involved), while exposure to GA had the least impact (40 affected genes). Figure 1 shows an overview of the extent of similarity among the responses of *L. plantarum* WCFS1 to *p*-CA, HXT, OLE, and RSV using the Venny 2.1 tool [30]. The assembled diagram shows the percentages of *L. plantarum* WCFS1 genes that overlap between the responses to these PPCs. When considering paired responses, the highest degree of coincidence was between OLE and *p*-CA where 25% and 32% of the genes, respectively, overlapped with respect to each other. The response to GA was also compared to the mentioned four phenolic compounds (not shown) displaying the best match with the response to *p*-CA (52.5% of the GA responsive genes were also regulated by *p*-CA). Comparative transcriptomic analysis of the specific responses to these individual PPCs revealed functional gene categories that highlighted the importance of adaptation of *Lactobacillus* cell surface properties, carbon and nitrogen metabolism, stress responsive pathways, and transport functions. The differentially expressed genes are incorporated and discussed in more detail as part of the sections below.

## 3. Oxidative Stress Responses Modulated by PPCs

Since antimicrobials usually feature the overproduction of reactive oxygen species (ROS) to exert their activity [3], oxidative stress responses can be expected when microbes interact with PPCs. Accordingly, *Lactobacillus* exposed to PPCs differentially expressed genes and proteins required to cope with oxidative stress (Table 1).

Methionine sulfoxide reductase, glutathione reductase, components of the thioredoxin (Trx)–thioreductase (TrxR) system, and a broad set of genes dedicated to methionine (Met) biosynthesis were transcriptionally upregulated in *L. plantarum* in response to *p*-CA [25]. This response is required to counter thiol-specific oxidative stress, and it is consistent with the pro-oxidative stress arising from *p*-CA autoxidation [31]. Methionine residues in proteins can be exploited by bacteria as ROS scavengers to protect proteins and lipids from oxidation [32,33]. Genes responsive to thiol-specific oxidative stress are induced by HXT in *L. plantarum* WCFS1, including methionine sulfoxide reductase, glutathione reductase, and a regulator of disulfide bond formation [29].

The stilbene RSV absolutely requires copper, a redox-active metal, to generate RSV-derived ROS. To counter this potential pro-oxidant behavior, *L. plantarum* responds to RSV by inducing a multicopper oxidase and CopR [27], a transcription factor that senses the copper status of the cytoplasm and controls the expression of copper export ATPases [34]. In agreement with this role, CopR has been suggested to play an important role in elevating resistance to H_2_O_2_ stress in *L. plantarum* [35]. In addition, RSV induces two zinc ABC transporters, a metal that competes with and antagonizes copper. One of these *L. plantarum* transporters (*lp_0464*) was also induced by OLE [28], and the corresponding *L. brevis* homologous permease (*lvis_0472*) was induced in cells exposed to ferulic acid [23] (Table 1). *L. plantarum* downregulates a Fe^2+^ transporter *mntH3* [36] upon HXT stress [29], which would diminish hydroxyl radical production arising from the Fenton reaction of this metal with H_2_O_2_. According to these profiles, management of the transport of transition metals participating in the Fenton-like chemistry seems a strategy used by *Lactobacillus* spp. to prevent an increase of the oxidative stress generated by PPCs.

Enzymes inactivating toxic oxygen radicals, which are part of the first defense against oxygen damage, were also implicated in the response to some PPCs (Table 1). The gene coding for the NADH peroxidase (*npr2*) was overexpressed by *L. plantarum* in the presence of OLE [28] or HXT [29]. A proteomic study revealed that the NPR2 enzyme was also upregulated in the presence of tannic acid [37]. Catalase (*kat*), which also detoxifies H_2_O_2_, was induced in *L. plantarum* upon HXT stress [29]. In addition, pyruvate oxidase (*pox*), an enzyme linked to oxidative stress resistance, was transcriptionally induced by OLE [28] or HXT [29].

The differential expression of the *cysE*–*metC*–*cysK* operon constituted a common response, except for GA, to PPCs in *L. plantarum* (Table 1). In *Lactococcus lactis* (a close relative of *L. plantarum*), the *metC*–*cysK* operon is well correlated with robustness toward oxidative stress [38]. In *L. plantarum*, the *cysE*–*metC*–*cysK* operon was downregulated upon *p*-CA [25] or RSV stress [27] but upregulated in the presence of OLE [28] or HXT [29]. The *metC* and *cysK* genes encode cystathionine-γ-lyase (CSE) and cystathionine-β-synthase (CBS), respectively, which are the microbial orthologs of the mammalian CSE and CBS. These are the most important H_2_S-generating enzymes in many bacteria [39], including *L. plantarum*, where CBS and CSE also produce H_2_S efficiently [40,41]. This gas has been shown to protect bacteria from the lethal effects of ROS formed from antibiotics [39].

Induction of the *L. plantarum cysE*–*metC*–*cysK* operon observed upon OLE or HXT stress indicates higher H_2_S generation. This profile was concurrent with the induction of NADH peroxidase, pyruvate oxidase, and catalase (Table 1). Protection against H_2_O_2_ provided by these enzymes and H_2_S agrees with the capacity of both phenolic compounds to generate H_2_O_2_.

In contrast, the *cysE*–*metC*–*cysK* operon was downregulated in the presence of *p*-CA [25]. Even though the *cysE*–*metC*–*cysK* operon is involved in methionine biosynthesis from cystathionine, *p*-CA still induced *L. plantarum* genes to rescue methionine biosynthesis via the alternative sulfhydrylase pathway [25], highlighting the role of this amino acid as an ROS scavenger in proteins.

## 4. Genotoxic Stress Responses

Some phenolic compounds could cause genotoxic stress as judged by the induction of genes and proteins that are induced by DNA damage. Tannic acid strongly overexpressed the RecA protein [37], which activates the SOS response upon DNA damage [42]. RSV induced up to eight genes of the pyrimidine biosynthetic route (required for the polymerization of DNA during repair) and genes linked to DNA repair mechanisms, including two *xerC* homologs, a DNA-3-methyladenine glycosylase II, and *mfd* and *pth*, a gene pair required to couple DNA repair and transcription [27]. The expression of genes encoding all components of the *L. plantarum* RNA degradosome was regulated by HXT [29], indicating that, along with DNA damage, RNA can also undergo oxidative stress in presence of some PPCs.

## 5. General Stress Pathways Activated in Response to PPCs

**Figure 2:**
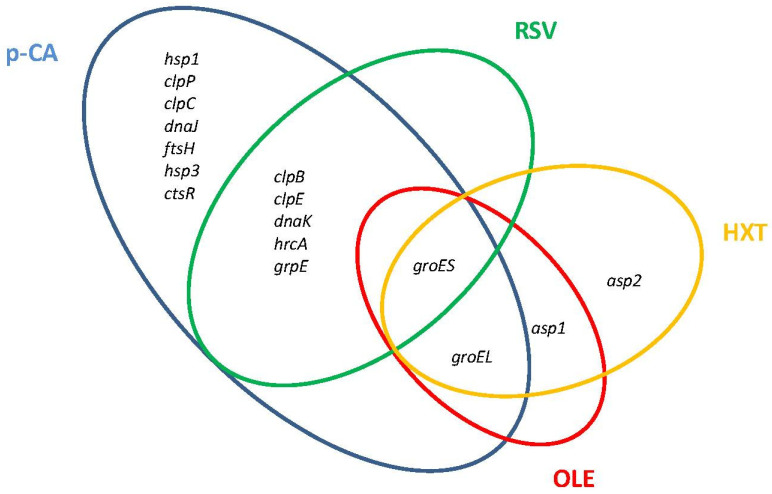
Venn diagram assembled from *Lactiplantibacillus plantarum* WCFS1 general stress-related genes differentially upregulated in response to individual plant phenolic compounds. *p*-CA, *p*-coumaric acid; RSV, resveratrol; OLE, oleuropein; HXT, hydroxytyrosol.

Major molecular chaperone and protease systems that accomplish essential functions to control protein quality under stress conditions are induced by *Lactobacillus* in the presence of PPCs (Figure 2).

The DnaK system (DnaK chaperone, co-chaperone DnaJ, and nucleotide exchange factor GrpE) is one of the chaperones involved in the molecular responses to PPCs. In *L. plantarum* WCFS1, components of the DnaK system were induced in the presence of *p*-CA or RSV (Figure 2). Another chaperone responsive to PPCs is the refolding GroES/GroEL system. GroES was transcriptionally induced in *L. plantarum* WCFS1 by *p*-CA, RSV, OLE, and HXT, while *groEL* was induced by these PPCs except by *p*-CA (Figure 2). Proteomic studies have shown that GroES is also notably induced at high tannic acid concentrations in *L. plantarum* [43], while GroEL is induced in response to tannic acid in *Lentilactobacillus hilgardii* [44] or the flavonol glycoside rutin in *Lactobacillus acidophilus* NCFM [45].

Several members of the Clp ATP family of proteases were induced by PPCs in *Lactobacillus*. Transcriptomic and proteomic studies have shown that the ClpP protease is induced by *p*-CA in *L. plantarum* WCFS1 [25], *Lacticaseibacillus casei* BL23 [24], and by rutin in *L. acidophilus* NCFM. The ClpC and ClpE proteases, which can associate with the ClpP protease in proteolytic complexes, are induced by *p*-CA in *L. plantarum* WCFS1 (Figure 2), whereas ClpE is induced by rutin in *L. acidophilus* NCFM. The expression of *clpE*, but not of *clpP*, was induced by RSV in *L. plantarum* WCFS1 [27] indicating that ClpE acts here as molecular chaperone [46]. In addition, *L. acidophilus* NCFM induced the two-component proteasome (HslV protease/HslU ATPase) in presence of rutin [45].

Other stress-related genes, including heat (*hsp*) and alkaline (*asp*) shock protein-encoding genes, also contribute to phenolic compound resistance in *Lactobacillus*. *Levilactobacillus brevis* cells exposed to ferulic acid (a hydroxycinnamic acid) [23] induced an *hsp* gene highly similar to the *hsp* genes induced by *p*-CA in *L. plantarum*. *L. acidophilus* NCFM also induced an hsp protein (GrpE) in the presence of rutin [45]. The *asp1* and *asp2* genes are induced in *L. plantarum* by OLE [28] and HXT [29], and the Asp1 protein is induced by tannic acid in *L. plantarum* [37]. Interestingly the homologous *L. plantarum asp2* gene of *L. casei* (*asp23*) contributes to gentamicin resistance in this microorganism [47].

Transcriptomic studies have revealed that the stringent response (SR), a conserved bacterial response that transcriptionally affects general stress responses [48,49], was involved in the molecular adaptation of *L. plantarum* WCFS1 to several PPCs. Several sets of genes owing to SR, including ribosome, nucleotide, and fatty-acid biosynthesis genes, were downregulated in *L. plantarum* WCFS1 by *p*-CA [25], OLE [28], or HXT [29]. In *L. brevis*, genes encoding proteins responsible for transcription, translation, and key proteins involved in cell division were strongly downregulated in the presence of ferulic acid [23]. Differential expression of *L. plantarum* genes coding for enzymes directly involved in the metabolism of (p)ppGpp (the alarmone assumed to trigger the SR), including small translational GTPases and (p)ppGpp synthases, were observed in the presence of HXT [29]. The (p)ppGpp metabolism is interconnected with GTP metabolism and regulates the intracellular concentrations of this nucleotide which, in turn, modulate the biosynthesis of the alarmone [49]. Genes related to GTP biosynthetic and GTP-consuming pathways were differentially regulated by *p*-CA [25], OLE [28], and HXT [29], indicating that GTP regulation is involved in the response to these PPCs, and that maintaining GTP levels within a range is essential for viability under different environmental conditions (Kriel et al. 2012).

It is worth noting that neither the chaperone/Clp machinery nor the SR were involved in the molecular response of *L. plantarum* to GA [26], indicating that the capacity to cause stress to *Lactobacillus* differs among PPCs.

## 6. Detoxification Mechanisms of PPCs in Lactobacillus

### 6.1. Metabolic Pathways for the Detoxification of PPCs

A means to overcome the toxicity of some PPCs is to metabolize them into less toxic compounds. The metabolic routes for the metabolism of PPCs that have been characterized at the molecular level to date in *Lactobacillus*, mainly hydroxycinnamic and hydroxybenzoic acids, were recently reviewed [50] and are not discussed here.

### 6.2. Drug Efflux and ABC-Transport Systems

The expression of *Lactobacillus* genes encoding several putative ABC-type and MFS drug extrusion systems are markedly modulated by different PPCs, which can contribute to directly counteract the toxic effects of these compounds.

As shown in Table 2, *L. plantarum* strongly induced two drug extrusion systems in presence of *p*-CA [25] or RSV [27]. One of these systems (*lp_0989* to *lp_0992*) is a multidrug efflux pump of the MFS superfamily. The *L. brevis* homolog of *lp_0991* (the multidrug transporter), *lvis_1917*, is also strongly induced by ferulic acid [23]. The other induced extrusion system is an ABC-type multidrug resistance (MDR) system encoded by genes *lp_2393 to lp_2395*. The permease of this system (*lp_2394*) is homologous to the LmrA MDR transporter from *Lactococcus lactis* [51], which efficiently extrudes drugs out of the cell.

Interestingly, an ABC-type transport system from *L. plantarum* encoded by the *lp_2739* and *lp_2740* genes was strongly downregulated by all PPCs compounds examined (*p*-CA, GA, RSV, HXT, and OLE) (Table 2). The permease of this system (*lp_2740*) displays a domain architecture that agrees with BceAB-type transporters [52], and it is homologous to the BceAB-like transporter OrABC from *L. casei* BL23 [24]. This type of Bce-like module plays crucial roles to sense and detoxify antimicrobial peptides (AMPs) [53] via import and subsequent intracellular enzymatic inactivation. However, the strong downregulation of this Bce-like module suggests that it does not take active part in resistance to PPCs, leaving its physiological significance to be established.

## 7. Metabolic Adaptations of *Lactobacillus* spp. to PPCs

### 7.1. Nitrogen Metabolism

**Figure 3:**
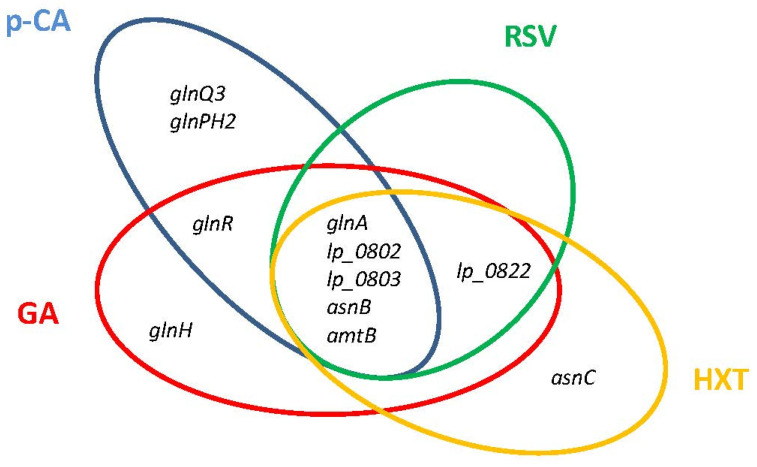
Venn diagram assembled from *Lactiplantibacillus plantarum* WCFS1 GlnR regulon genes involved in nitrogen metabolism that are differentially downregulated in response to individual plant phenolic compounds. *p*-CA, *p*-coumaric acid; RSV, resveratrol; HXT, hydroxytyrosol; GA, gallic acid.

The *L. plantarum* transcriptome responses to GA [26], RSV [27], HXT [29], and *p*-CA [25] share a conserved profile that entail the downregulation of genes involved in nitrogen metabolism (Figure 3). These genes, all under the control of GlnR regulator [54], include the glutamine (Gln) synthetase (*glnA*), aspartate ammonia lyase which produces ammonium (*asnB*), a Gln ABC transporter (*lp_0802* and *lp_0803*), and the ammonium transporter protein (*amtB*). The observed profile agrees with a tight control of intracellular ammonia levels, the previously proposed regulatory function of GlnR in *Lactobacillus*, which would be achieved by constraining the import of ammonia/amino-containing compounds and controlling the production of intracellular ammonia and glutamine [55]. The regulation of nitrogen metabolism triggered by PPCs could contribute to control the bacterial access to host glutamine, which is a hub for nitrogen metabolism in plants and bacteria [56] and can be important for plant–bacterial combinations. It is to note that OLE, a PPC proposed to conduct a close association between *L. plantarum* and Oleaceae plant hosts [28], does not modulate the GlnR regulon which could facilitate nitrogen transfer from the plant to the microbial symbiont, an essential requirement for symbiotic relationships. This knowledge opens the possibility to use suitable PPCs to better control the bacterial access to host glutamine. In the case of some human pathogens, preventing access to host glutamine is required to attenuate its virulence [57,58].

### 7.2. Sugar and Energy Metabolism

*L. plantarum* altered the expression of genes or proteins involved in the transport and metabolism of different carbon sources in response to PPCs.

*L. plantarum* decreased the expression of glucose permease and increased the expression of genes involved in the malolactic fermentation (MLF) pathway in the presence of *p*-CA. This metabolic adaptation generates energy via chemiosmotic mechanisms and increases the intracellular pH to reduce acid stress. A similar transcriptomic reprogramming was observed during the fermentation by *L. plantarum* of vegetable and fruit juices rich in hydroxycinnamic acids [59,60]. Upregulation of genes involved in the MLF pathway has also been observed during the response of *L. brevis* to ferulic acid [23].

Genes encoding transketolase (*tkta*), phosphoglycerate mutase (*pgm*), and phosphoketolase (*xpkA*) were upregulated under *p*-CA stress [25]. This profile suggests accumulation of glycolytic intermediates such as 3-phosphoglycerate and 2-phosphoglycerate, a metabolic adaptation characteristic of carbohydrate starvation conditions that is supported by *uspA* induction, which encodes a universal stress protein leading to a continuous growth-arrest state [61]. The Pgm and UspA proteins were also induced under tannic acid stress [37], suggesting similar metabolic adaptations.

OLE transcriptionally reprogrammed the expression of transporters from the phosphotransferase (PTS) system of *L. plantarum* [28] including the induction of the mannitol and fructose PTS systems which are the main soluble components of olive source and sink tissues. This expression profile would promote sugar transfer from the host and support the proposed role of OLE as a signaling molecule in the association between *Lactobacillus* and the plant host [28].

*L. plantarum* WCFS1 encodes an *N*-acetyl glucosamine (GlcNAc) transporter (*nagE*) that is downregulated in presence of *p*-CA, OLE, and RSV [25,27,28]. However, the expression of *nag* genes involved in GlcNAc catabolism was not affected by these PPCs. The concurrent downregulation of genes involved in the production of cell-wall and capsular polysaccharide constituents suggests that *nagE* downregulation is related to cell-wall remodeling, as GlcNAc may act as a building block for biosynthesis of these cellular components.

Unlike *p*-CA, OLE, and RSV, the transcriptomic signature of GA showed increased expression of genes required for GlcNAc utilization, including those coding for glucosamine-6-P isomerase (*nagB*) and a putative PTS transporter of the [GlcNAc]_2_ disaccharide (*lp_2954*) suggesting that GA promotes GlcNAc utilization by *L. plantarum* WCFS1 [26].

### 7.3. Gallic Acid: A Source of Chemiosmotic Energy to Lactobacillus

DNA microarray experiments and physiological analysis established that GA triggered a specific metabolic adaptation in *L. plantarum*: the transport and catabolism of GA was self-inducible and supplied chemiosmotic energy to this microorganism [26]. GA triggered a huge transcriptional induction of the GA transporter (*gacP*) and the three gallate decarboxylase subunits (*lpdB*, *lpdC*, *lpdD)* [26]. The uptake of monoanionic GA by *gacP* was coupled to the extrusion of the GA decarboxylation product, uncharged pyrogallol [26]. Membrane potential and internal pH measurements showed that the transport process and the cytosolic H^+^ ions consumed during gallic acid decarboxylation by the gallate decarboxylase generated ∆pH and ∆Ψ gradients, thus increasing the proton motive force (PMF) over the cell membrane and the intracellular pH to reduce acid stress [26].

## 8. Membrane and Cell-Wall Modifications in Response to PPCs

### 8.1. Cell-Wall Modifications

*Lactobacillus* regulates the expression of genes and proteins involved in the biosynthesis of all types of macromolecular components of the cell envelope in response to PPCs, including peptidoglycan (PG), teichoic acids (TAs), and capsular polysaccharides (cps).

Modification of the biosynthesis of PG precursors is a known adaptation to stress conditions in Gram-positive bacteria [62,63]. Some PPCs such as tannic acid inflict injuries in the cell wall leading to rougher cell surfaces and leakages [64]. To circumvent the inhibitory effects of tannic acid, *L. plantarum* modifies its profile of PBPs (penicillin-binding proteins) (64), which are enzymes known to play a key role in PG biosynthesis. In addition, *L. plantarum* respond to TA aggression by overproducing LdhD (d-lactate dehydrogenase) and DapF (diaminopimelate (DAP) epimerase), two proteins required for the biogenesis of the PG precursors d-lactate and *meso*-diaminopimelic acid (meso-DAP), respectively [37]. Similarly, *L. hilgardii* and *L. acidophilus* NCFM overexpressed LdhD under tannic acid [44] or rutin stress [45], respectively. In addition to LdhD, *L. acidophilus* NCFM overexpressed GlmU in the presence of rutin, this enzyme catalyzing the last steps of UDP-GlcNAc, one of the cell-wall peptidoglycan precursors.

*p*-CA injures and leads the bacterial membrane to become leaky [65]. The transcriptomic response of *L. plantarum* to *p*-CA [25] revealed overexpression of *aad* (d-alanyl-d-alanine dipeptidase) and *hicD2 (*d-hydroxyisocaproate dehydrogenase), which reportedly modify the biosynthesis of PG precursors [66]. This profile coincided with a marked decrease in the expression of genes encoding putative lytic glycosyl-transferases (*lp_0302*, *lp_3014*, *lp_3015*) and a muropetidase (*lp_3421*), all encompassed within the peptidoglycan hydrolase (PGH) complement of *L. plantarum* WCFS1 [67]. This transcriptome reprogramming does point to PG modification, reduction in PG turnover, and repression of cell lysis to counter the effects of *p*-CA [25].

The production levels and structure of wall teichoic acids (WTA) and lipoteichoic acids (LTA) from *L. plantarum* were likely affected by the presence of some PPCs as judged by the altered expression of genes and proteins responsible for their production. TagE6 (poly(glycerol-phosphate) α-glycosyltransferase), an enzyme involved in glucosyl substitution of poly(ribitol-5-P) WTA [68], was induced after exposure to tannic acid [37] and transcriptionally overexpressed in response to OLE [28] or HXT [29].

Induction of genes of the *tarIJKL* locus is a genetic marker of WTA backbone alditol switching in *L. plantarum* [68] as it is responsible for poly(ribitol-5-P) WTA production, an alternative WTA variant to the wild-type glycerol-containing backbone. The *tarIJK* and the *tarK* genes are transcriptionally induced in response to OLE [28] and HXT [29], respectively, suggesting that WTA backbone alditol switching occurs in response to these PPCs. Downregulation of *tagD2* (*lp_1248*), which CDP-activates glycerol-P, was also observed in presence of HXT, further supporting WTA backbone alditol switching. In addition, downregulation of *gtca1* which encodes an LTA glycosylation protein suggests changes in the decoration of LTAs exposed to HXT.

The capsular polysaccharide (*cps*) genes encode the enzymatic machinery involved in the different steps of the repeating unit synthesis, export, and polymerization of these macromolecules in lactic acid bacteria [69,70]. The decreased expression of *cps* genes was a common transcriptome response of *L. plantarum* WCFS1 to *p*-CA [25], RSV [27], and OLE [28]. These genes are organized in four *cps* gene clusters distributed along the genome of this microorganism. In the presence of *p*-CA, up to 16 out of all 36 *cps* genes that were encompassed within *cps1*, *cps3*, and *cps4* gene clusters were downregulated. In the presence of OLE, 21 out of 36 *cps* genes were downregulated including genes from all *cps1*, *cps2*, *cps3*, and *cps4* clusters. In the presence of RSV, three genes of the *cps4* gene cluster and genes involved in the transport and metabolism of precursors for *cps* biosynthesis (mannose, glucosamine-6-P, and GlcNAc) were downregulated. It is also to note that CPS was not detectably accumulated over the outer surface of *L. plantarum* WCFS1 exposed to tannic acid [64].

### 8.2. Membrane Modifications

Proteomic and DNA microarray experiments revealed that PPCs markedly regulate the expression of genes and proteins involved in membrane lipid biosynthesis. Most *L. plantarum* genes from the fatty-acid (FA) biosynthesis (*fab*) locus were downregulated in the presence of *p*-CA [25] or OLE [28]. Decreased expression of the *fab* locus is indicative of membrane modifications in its FA composition, which is a common strategy in *Lactobacillus* spp. to counter different types of stress conditions [71,72,73,74]. *L. casei* BL23 or *L. brevis* upregulated one protein of the FAB pathway in response to *p*-CA [24] or ferulic acid [23], respectively.

Proteomic and transcriptomic studies have shown that *L. plantarum* WCFS1 downregulates a cyclopropane-fatty-acyl-phospholipid synthase (Cfa2) in the presence of TA [37] or OLE [28]. Cfa2 was also downregulated in *E. coli* in response to cranberry polyphenols [75] or naringerin [76]. This adaptation alters the cyclic to saturated membrane FA ratio (CFA/SFA) which influences the membrane fluidity. Combined downregulation of the *fab* locus and *cfa2* suggests changes in membrane FA composition to counteract the damage inflicted by some PPCs in the *Lactobacillus* cell membrane.

Membrane phospholipid remodeling can also be involved in the adaptation of *Lactobacillus* to PPCs. The OLE-responsive transcriptome showed a set of genes coordinately expressed by *L. plantarum* in ways to increase the synthesis of *sn*-glycerol-3-P, the obligated glycerophospholipid precursor [28]. This modification may be important in the adaptation of *Lactobacillus* spp. to PPCs since membrane phospholipid alterations are crucial for bacterial adaptability to environmental stress [77].

Since a high number of genes encoding membrane proteins were overexpressed during the responses of *L. brevis* to ferulic acid [23] and *L. plantarum* to *p*-CA [25], membrane crowding with proteins has been proposed to stabilize the *Lactobacillus* membranes against hydroxycinnamic acids.

Genes encoding transporters of compatible solutes including glycine-betaine/carnitine/choline, glycerol, trehalose, or GABA were downregulated upon exposure of *L. plantarum* to *p*-CA [25] or HXT [29]. This can contribute to stabilize the membrane, as previously reported for trehalose in *L. acidophilus* [78]. Membrane damage can cause proton motive force (PMF) dissipation [79,80]. In *Lactobacillus* spp., this disturbance can be in part compensated for by an increased abundance of F_1_/F_0_ ATPase that pumps protons to the outside environment [81,82]. Upregulation of the *L. plantarum* F_1_/F_0_ ATPase components in the presence of HXT [29] suggests that this PPC causes PMF dissipation.

## 9. Role of PPCs in the Molecular Adaptation of *Lactobacillus* to Host Environments

PPCs can be released by plants to enable bidirectional interfaces with the environment. Within these interactions, the microbe signaling mediated by PPCs can be crucial for plant robustness. A known example is the microbe signaling by plant isoflavonoids to transcriptionally activate bacterial key genes for nodule formation that stimulate plant growth [83,84]. Benzoxazinoids can also acts as mediators of plant health by modulating the soil microbiome [85].

These cases illustrate the ability of some PPCs to shape the structure and function of plant host–microbial communities. This capacity may operate not only in setting up phytomicrobiomes but also to shape the microbiome structure and function across the animal kingdom given that PPCs are consumed in the diet. Because the host microbiome markedly influences the host phenotype, it is necessary to understand the direct effects of PPCs on microbes and the influence of PPCs on host–microbial interactions to establish a causal relationship between PCs exposure and effects on the host.

Molecular details on how PPCs influence the survival and function of *Lactobacillus* spp. in host environments remain largely unknown. This section touches on a few approaches that reveal how individual PPCs can modulate the expression of molecular traits from *Lactobacillus* spp. reportedly involved in the adaptive response to host environments or that have documented functions in the host–*Lactobacillus* molecular dialog.

### 9.1. Regulation of Molecular Functions Involved in the Survival of Lactobacillus to Gastrointestinal (GI) Tract Stress

Lactobacilli undergo different stress conditions during the passage through the GI tract. Interestingly, genes coding for different molecular functions involved in the survival of *L. plantarum* to the GI tract conditions were also responsive to PPCs (Table 3).

*L. plantarum* WCFS1 submitted to an orogastric–intestinal (OGI) simulator [86] induced a set of proteases (*clpB*, *clpE*, *clpP, fstH*), molecular chaperones (*groEL*, *dnaK*), and heat-shock proteins (*hsp1*, *hsp2*, *hsp3*). Figure 2 shows that all these genes were also induced by *p*-CA while some of them were also responsive to RSV, HXT, and OLE.

High osmolarity and contact with bile salts in the duodenum lead *L. plantarum* to induce a relatively high number of genes involved in cell envelope functions [87,88]. *L. plantarum* triggers similar responses when it contacts with tannic acid, *p*-CA, or OLE, including the regulation of the expression of genes involved in the biosynthesis of cell envelope constituents or that are major actors in cell-wall turnover (Table 3). Multidrug transporters may also play an important role for bile tolerance in *Lactobacillus* spp. [82,89]. In this regard, *p*-CA and RSV markedly induced *L. plantarum* genes encoding an efflux pump (*lp_0990*-*lp_0992*) that is also induced in the small intestine and proposed to be involved in the extrusion of bile salts [90] (Table 3). To counteract the high osmolarity stress in the small intestine, *L. plantarum* involves transporters of compatible solutes including glycine-betaine/carnitine/choline [91], which are also responsive to *p*-CA and HXT (Table 3).

In addition to acidic and high-osmolarity conditions, bacteria face oxidative stress at the mucosal surface of the colon [92]. As mentioned above, *Lactobacillus* activates different mechanisms to counter oxidative stress in the presence of PPCs (Table 3) which can cross-protect these bacteria in the colon. 

In addition to these molecular mechanisms, more functions related to the survival of *Lactobacillus* in the GI tract can be individually controlled by *p*-CA, GA, TA, or RSV, as described in the next sections.

#### 9.1.1. Gallic Acid

GA strongly induces the expression of *lp_2940*, a crucial gene for the persistence and survival of *L. plantarum* WCFS1 in the GI tract [93]. GA also induces genes involved in GlcNAc utilization. This amino sugar is part of the human intestinal mucus glycoproteins and can be used as a carbon source by *Lactobacillus*, particularly under bile stress [82]. The increased capacity to use GlcNAc together with the overexpression of genes coding for tannase and GA metabolism (see above) may confer competitive advantages to *L. plantarum* in the GI tract where nutrients are not in constant supply.

#### 9.1.2. Tannic Acid

Four genes markedly induced in the GI tracts of human and mouse (*argG*, *copA*, *ram2*, and *lp_2940*) were upregulated upon exposure to tannic acid [64]. From these, *copA* and *lp_2940* [90,94] are crucial for the persistence and survival of *L. plantarum* in the digestive tract. In addition, the penicillin-binding PBP2A protein, a biomarker negatively related with GI survival [95], was inactivated at the post-translational level by tannic acid suggesting enhanced GI survival reportedly associated with the inactivation of this function [64].

#### 9.1.3. *p*-Coumaric Acid (*p*-CA)

*L. plantarum* treated with *p*-CA overexpressed a gene set also induced when this microorganism was perfused in the small intestine [90], which includes an alkaline shock protein (*asp2*), universal stress protein (*uspA*), several chorismate biosynthesis genes, and an amino-acid ABC transporter (*lp_1744–1746*).

#### 9.1.4. Resveratrol

*L. plantarum* overexpressed the *lp_3368* gene in the presence of RSV. This gene encodes a multidrug transporter potentially able to use deoxycholate as a substrate [96], a function consistent with its location in the vicinity of *bsh3*, a bile salt hydrolase-encoding gene [97]. Transport of secondary bile acids that are produced by BSH activity can govern bacterial fitness and host colonization [98], as well as alter host physiology [99].

The capacity of PPCs to modulate the expression of genes related to GI tract survival may be of utility to improve fitness of lactobacilli in this niche, more in view that gut robustness of individual strains may depend on differential gene expression levels rather than on the presence or absence of conserved genes [100].

### 9.2. Remodeling of the Cell Envelope Induced by PPCs: Potential Impact on the Communication Capacities of Lactobacillus with the Host

The *Lactobacillus* cell envelope is a source of molecules that act as key probiotic ligands. These molecules are known to interact with host receptors inducing signaling pathways that result in probiotic effects [87,91,92,101]. As mentioned above, the biosynthesis of cell-wall constituents (peptidoglycan, teichoic acids, polysaccharides, and proteins) is subjected to regulation by PPCs. Hence, PPCs emerge as new potential modulators of the cell surface properties from *Lactobacillus* spp.

#### 9.2.1. Capsular Polysaccharides

The capacity of *L. plantarum* to synthesize CPS was markedly downregulated in the presence of different PPCs (see above and Table 4). CPSs may shield adhesion proteins and microorganism-associated molecular patterns (MAMPs) interacting with pattern recognition receptors (PRRs) of dendritic cells, which play key roles in innate and adaptive immunity. Accordingly, CPS downregulation has been found to be required for optimal adherence of *L. rhamnosus* GG to intestinal epithelial cells [102] (Lebeer et al. 2009), and deletion mutants in the four *cps* clusters of *L. plantarum* WCFS1 alter its immunomodulatory capacities by increasing the exposure of bacterial MAMPs to their host receptors to induce signaling cascades [69,103]. Therefore, decreased expression of *cps* genes driven by the exposure to some PPCs is likely to optimize the immunomodulatory and adhesion capacities of *Lactobacillus.*

#### 9.2.2. Teichoic Acids

The different biochemical properties (chain length, backbone composition, or degree of glycosyl substitution) and production levels of cell wall teichoic acids (WTA) and lipoteichoic acids (LTA) are directly related to the inmunodulatory capacity of *Lactobacillus* spp. [104] and severely impact its capacity to communicate with their hosts [68]. The expression of several enzymes implicated in the biosynthesis or modification of *L. plantarum* teichoic acids that are associated with altered host responses, including TagE6, Tag D2, enzymes encoded by the *tarIJKL* locus, and the LTA glycosylation protein encoded by the *gtca*1 gene, were differently regulated by various PPCs, mainly OLE and HXT (Table 4).

According to these observations, exposure to these PPCs is expected to modify the signaling ability of *L. plantarum* and its capacity to communicate with their hosts. This notion is strongly supported in the case of OLE [28] as exposure to this PPC also modifies the expression of other documented immunomodulators from this microorganism, including components of the plantaricin and LamBDCA quorum-sensing systems [105,106,107,108].

#### 9.2.3. Surface and Moonlighting Proteins

Analysis of differential whole-cell and surface proteomes of the probiotic *L. acidophilus* NCFM strain revealed that ferulic acid, reveratrol, tannic acid, and caffeic acid varied the abundance of moonlighting proteins engaged in adhesion [109] (Table 4). These profiles were associated with variations in the adhesive capacity of this strain triggered by these PPCs. The abundance of some of these moonlighting proteins including EF-P, pyruvate kinase, and EF-Tu correlated well with binding capabilities to HT-29 cells triggered by RSV or caffeic acid. Although adhesion capacities have not been tested in presence of rutin, *L. acidophilus* NCFM also overexpresses EF-P and pyruvate kinase (as well as GAPDH and EF-G) in the presence of this flavonol glycoside [45]. In this regard, the same positive correlation between rutin and *L. acidophilus* adhesive capacity could be expected.

However, it must be noted that abundance of GAPDH or EF-G did not fit with the good adhesion capacities of this strain stimulated by RSV or TA, respectively [109]. The expression of two moonlighting proteins implicated in adhesion, EF-GreA [110] (upregulated) and LuxS [111] (downregulated), was also of different tendency in response to tannic acid in *L. plantarum* WCFS1 [37] (Table 4). Overall, these results show that abundance of moonlighting proteins with potential adhesion capacity cannot always be directly correlated with improved adhesion induced by these PPCs, indicating that mutant approaches are necessary to complete these studies. Other candidate proteins with moonlighting functionality have been implicated in adhesion to host cells [112,113,114]. Among these, the oligopeptide-binding protein Opp [115] or some molecular chaperones [116,117,118], are markedly induced in presence of some PPCs. The *oppA* gene is markedly overexpressed in the presence of *p*-CA or OLE (Table 4). GroEL and small heat-shock proteins (HSPs) are induced by several PPCs (Table 4) and even though these proteins participate in the binding of *L. johnsonii* and *L. plantarum* WCFS1 to host cells, respectively, adhesion assays in the presence of PPCs and mutant approaches are also required to show which among these moonlighting proteins induced by specific PPCs improve the adhesion to host cells.

The *msa* gene from *L. plantarum* encodes a mannose-specific adhesion factor [67] that is overexpressed in the presence of RSV (Table 4). Since improved adhesion to mannose-containing intestinal cells is believed to be important for the health-promoting effects of probiotics [119], induction of *msa* by RSV may help in improving the adhesion capacity and probiotics effects of *L. plantarum*.

## 10. Concluding Remarks

Exposure to plant phenolic compounds leads to changes in the composition of plant and animal host microbiomes which can decisively contribute to host fitness and health. However, our understanding of the molecular mechanisms underlying the direct effects of PPCs on microbes, as well as on host–microbe interactions, is still largely unexplored, in terms of compounds, microbes, and hosts examined. These insights are necessary to substantiate a causal nexus between exposure to PPCs and its effects on microbes and the host.

Here, molecular research into the interaction between *Lactobacillus* spp. and individual PPCs was reviewed (Table 5). A variety of mechanisms are induced by PPCs to increase the oxidative stress response in these microorganisms which highlights the antimicrobial nature of these compounds. These responses, together with the induction of general stress responses, revealed that these compounds are perceived, but not always, as stressors by these microorganisms. To cope with this selective pressure, *Lactobacillus* relies, in addition to known metabolic pathways for PPC detoxification, on efflux pumps to extrude, at least, hydroxycinnamic acids and stilbenes out of the cell. Furthermore, exposure to different PPCs variably adjusts the capacity of lactobacilli for carbohydrate acquisition and metabolism. The adaptation of nitrogen metabolism in to different PPCs is conserved and oriented to control the import and production of ammonia/amino-containing compounds. These metabolic adaptations, together with the induction of oxidative and general stress responses, are important ecological fitness determinants that leave *Lactobacillus* better able to cope with the stress encountered in the host environment.

The biosynthetic pathways for macromolecular components of the cell wall, which play key physiological roles in the communication of lactobacilli with the host environment and directly participate in host signaling, were markedly modulated by PPCs. In addition, some PPCs regulate the expression of specific molecular components with documented immunomodulatory capacities. PPCs, thus, emerge as new potential modulators of the cell surface and immunomodulatory properties of *Lactobacillus* spp. This finding opens new promising ways to target modifications with these natural products of cell surface properties that are associated with the physiological status of the host. The increased understanding of the *Lactobacillus*–PPC interaction provides molecular criteria to rationally improve the effector capacities and increase the success rates when these microorganisms are administered to improve plant growth or to benefit animal host health.

## Figures and Tables

**Table 1:** Genes and proteins of *Lactiplantibacillus plantarum* WCFS1 induced by plant phenolic compounds that are responsive to oxidative stress.

Phenolic Compound	Molecular Mechanism and Actors Involved			References
	Enzymatic Inactivation of O_2_ Radicals	Repair of O_2_ Radical-Induced Damage	Modulation of Metal Transport Participating in the Fenton-Like Chemistry	
Tannic acid	Npr2	RecA		[37]
*p-*coumaric acid		*gshR2* *trxA1, trxA3, trxB1* *msrA2*, *msrA3*, *msrA4* *tpx* *CBS*,* CSE*		[25]
Resveratrol		*xerC* *mfd* *pth* *CBS, CSE*	*copR* *lp_0355* (multicopper oxidase) *lp_3302, lp_0463* (zinc ABC transporters)	[27]
Oleuropein	*npr2* *pox5 (lp_3589)*	*gshR2* *msrA4* *lexA* *CBS, CSE*	*lp_0463, lp_0464* (zinc ABC transporter)	[28]
Hydroxytyrosol	*npr2* *kat (lp_3578)* *pox4 (lp_3587)*	*msrA3* *gshR4* *lp_0858* *CBS, CSE*	*lp_2992 (mntH3)* (iron transporter)	[29]

Black font: upregulated genes or proteins. Red font: downregulated genes.

**Table 2:** Drug efflux and ABC transport systems of *Lactiplantibacillus plantarum* WCFS1 differentially expressed in presence of plant phenolic compounds.

Phenolic Compound	Transporter Type	Locus Tags ^a^ and Fold Change ^b,c^				References
	ABC (BceAB-like)					
		*lp_2739*	*lp_2740*			
GA		−7.2	−6.7			[26]
*p*-CA		−6.4	2.0			[25]
RSV		−12.4	−2.7			[27]
OLE		−20.4	−16.6			[28]
HXT		−4.7	−3.8			[29]
	MFS					
		*lp_0989*	*lp_0990*	*lp_0991*	*lp_0992*	
*p*-CA		3.0	19.5	17.5	16.7	[25]
RSV		5.8	5.6	5.5	5.7	[27]
	LmRA-like					
		*lp_2393*	*lp_2394*	*lp_2395*		
*p*-CA		10.2	8.7	9.8		[25]
RSV		3.8	4.6	6.5		[27]

^a^ Designated gene number for the annotated *L. plantarum* WCFS1 genome; ^b^ fold change refers to growth in MRS supplemented with the corresponding PPC relative to growth in MRS without supplement; ^c^ FDR ≤ 0.05; *p* < 0.05.

**Table 3:** Genes and proteins of *Lactiplantibacillus plantarum* WCFS1 associated with improved survival against gastrointestinal tract-induced stress that are differentially expressed by plant phenolic compounds.

Locus Tags		PPC Effector (Ref.) ^a^	Effect	Phenotype Involved (Ref.) ^b^
**In vitro orogastric–intestinal (OGI) survival**				
*lp_1903*	*clpB:* ATP-dependent Clp protease ATP-binding subunit	*p*-CA [24], RSV [27]	+	In vitro OGI survival [86]
*lp_1269*	*clpE:* ATP-dependent Clp protease ATP-binding subunit	*p*-CA, RSV	+	In vitro OGI survival
*lp_0786*	*clpP:* ATP-dependent Clp protease, protease subunit	*p*-CA	+	In vitro OGI survival
*lp_0547*	*ftsH: cell division protease*	*p*-CA	+	
*lp_2029*	*hrcA:* heat-inducible transcriptional repressor	*p*-CA	+	In vitro OGI survival
*lp_0728*	*groEL:* chaperonin groEL	*p*-CA, HXT [29], OLE [28]	+	In vitro OGI survival
*lp_0727*	*groES:* chaperonin groES	*p*-CA, HXT, RSV, OLE,	+	In vitro OGI survival
*lp_2027*	*dnaK*: molecular chaperone	*p*-CA, RSV	+	In vitro OGI survival
*lp_0129*	*hsp1*: small heat-shock protein 1	*p*-CA	+	In vitro OGI survival
*lp_3352*	*hsp3*: small heat-shock protein 3	*p*-CA	+	In vitro OGI survival
**Bile resistance**				
*lp_2365* *lp_2367* *lp_2368*	*atpG*: F-type H+-transporting ATPase subunit gamma *atpH*: F-type H+-transporting ATPase subunit delta *atpF*: F-type H+-transporting ATPase subunit b	HXT	+	Bile resistance [91]
*lp_1253*	*gshR2*: glutathione reductase	*p*-CA, OLE	+	Bile resistance
*lp_0254*	*cysE*: serine *O*-acetyltransferase	HXT, OLE	+	Bile resistance
*lp_0255*	*metC1*: cystathionine beta-lyase	HXT, OLE	+	Bile resistance
*lp_0256*	*cysK*: cysteine synthase	HXT, OLE	+	Bile resistance
**Gastro-intestinal tract (GIT) passage**				
*lp_2940*	cell surface protein precursor, LPXTG-motif cell-wall anchor	TA [64], GA [26]	+	Human/mouse GIT passage [95]
*lp_3055*	*copA*: copper-transporting ATPase	TA [64]	+	Human/mouse GIT passage [95]
*lp_0775*	*argG*: argininosuccinate synthase	TA [64]	+	Mouse GIT passage [88,90]
*lp_3473*	*ram2*: alpha-l-rhamnosidase	TA [64]	+	Mouse GIT passage [90]
PBP2A	transpeptidase–transglycosylase (penicillin binding protein 2A)	TA [64]	-	GIT-robustness [97]
*lp_1835*	*msrA2* protein-methionine-*S*-oxide reductase	*p*-CA	+	Maintenance in gut ecosystems [95]
*lp_1836*	*msrA3* protein-methionine-*S*-oxide reductase	HXT, *p*-CA	+	Maintenance in gut ecosystems [95]
*lp_1979*	*msrA4* protein-methionine-*S*-oxide reductase	OLE, *p*-CA	+	Maintenance in gut ecosystems [95]
*lp_0991*	multidrug transport protein, major facilitator superfamily (MFS)	*p*-CA, RSV	+	Human small intestine passage [92]
*lp_0930*	*asp2*: alkaline shock protein	*p*-CA	+	Human small intestine passage [92]
*lp_2034* *lp_2035* *lp_2037*	*tyrA*; prephenate dehydrogenase *aroE*; 3-phosphoshikimate 1-carboxyvinyltransferase *aroF*; chorismate synthase	*p*-CA *p*-CA *p*-CA	+ + +	Human small intestine passage [92]
*lp_1744* *lp_1745* *lp_1746*	d-methionine ABC transporter, ATP binding protein d-methionine ABC transporter permease d-methionine ABC transporter, substrate-binding protein	*p*-CA *p*-CA *p*-CA	+ + +	Human small intestine passage [92]
**Other mechanisms**				
**Oxidative stress**				
*npr2* *kat* *pox* *trxA1*, *trxA*; *trxB1* *tpx* *gshR4* *lp_0858*	NADH-peroxidase catalase pyruvate oxidase thioredoxin; thioredoxin reductase thiol peroxidase glutathione reductase redox protein, peroxiredoxin	*p*-CA, OLE, HXT HXT OLE, HXT *p*-CA *p*-CA HXT HXT	+ + + + + +	Resistance against oxidative stress at mucosal surface of the colon (putative) [94]
**Peptidoglycan remodeling**				
LDH-D	d-lactate dehydrogenase	TA [37] Rutin [45]	+ +	Maintaining integrity of the cell envelope under GIT stress (bile, osmotic and acid stresses) (putative) [87]
DAPF	diaminopimelate (DAP) epimerase	TA [37]	+	
GLMU	UDP-*N*-acetylglucosamine pyrophosphorylase	Rutin [45]	+	
*aad*	d-alanyl-d-alanine dipeptidase	*p*-CA	+	
*hicD2*	d-hydroxyisocaproate dehydrogenase	*p*-CA	+	
*lp_0302;lp_3014; lp_3015*	lytic transglycosylases	*p*-CA	+ −	
**Compatible solutes**				
*lp_3324* *glpF4* *pts5ABC*	glycine betaine/carnitine/choline transport protein glycerol uptake facilitator protein PTS system specific- trehalose transporter	*p*-CA, HXT *p*-CA HXT	− − −	Osmoprotection against osmotic stress in the GIT (putative) [87,94]

^a^ Reference describing the regulation of the molecular actor (gene or protein) by the plant phenolic compound. *p*-CA (*p*-coumaric acid), RSV (resveratrol), OLE (oleuropein), HXT (hydroxytyrosol), TA (tannic acid); ^b^ reference describing the phenotype associated with the molecular actor (gene or protein); +, upregulation; −, downregulation.

**Table 4:** Genes and proteins of *Lactobacillus* involved in the interaction with host cells that are differentially expressed by plant phenolic compounds.

Locus Tags		Species	PPC Effector (Ref.) ^a^	Effect	Phenotype Involved (Ref.) ^b^
**Surface polysaccharide biosynthesis**					
*lp_1177 through lp_1185*	*cps1A-I:* polysaccharide biosynthesis gene cluster 1 proteins	*L. plantarum*	*p*-CA [25]	−	Reduced SPS levels [69,104,105]
*lp_1220-lp_1221*	*cps3DE*: polysaccharide biosynthesis gene cluster 3 proteins		*p*-CA	−	Reduced SPS levels
*lp_2101-lp_2103; lp_2106-lp_2108*	*cps4HGF*: polysaccharide biosynthesis gene cluster 4 proteins		*p*-CA	−	Reduced SPS levels
*lp_2101-lp_2102; lp_2106*	*cps4HG; cps4C*: polysaccharide biosynthesis gene cluster 4 proteins		RSV [27]	−	Reduced SPS levels
*lp_1180; lp_1184-lp_1185*	*cps1D; cps1H-I:* polysaccharide biosynthesis gene cluster 1 proteins		OLE [28]	−	Reduced SPS levels
*lp_1197- lp_1198*	*cps2AB*: polysaccharide biosynthesis gene cluster 2 proteins		OLE	−	Reduced SPS levels
*lp_1201- lp_1203; lp_1207*	*cps2EFG; cps2K*: polysaccharide biosynthesis gene cluster 2 proteins		OLE	−	Reduced SPS levels
*lp_1215; lp_1222-lp_1224*	*cps3A; cps3FG*: polysaccharide biosynthesis gene cluster 3 proteins		OLE	−	Reduced SPS levels
*lp_2099-lp_2104;* *lp_2106-lp_2108*	*cps4JIH; cps4GFE*: polysaccharide biosynthesis gene cluster 4 proteins		OLE	−	Reduced SPS levels
**Teiochoic acid biosynthesis**					
*lp_2844*	*tagE6*: poly(glycerol-phosphate) alpha-glucosyltransferase	*L. plantarum*	HXT [29], OLE	+	Glycosyl substitution of WTA [68]
TagE6	Poly(glycerol-phosphate) alpha-glucosyltransferase		TA [37]	+	Glycosyl substitution of WTA [68]
*lp_1816*	*tarI*: d-ribitol-5-phosphate cytidylyltransferase		OLE	+	Synthesis of alternative WTA variants [68]
*lp_1817*	*tarJ*: ribitol-5-phosphate 2-dehydrogenase		OLE	+	Synthesis of alternative WTA variants [68]
*lp_1818*	*tarK*: ribitolphosphotransferase		OLE, HXT	+	Synthesis of alternative WTA variants [99]
*lp_1372*	*gtca1*: teichoic acid glycosylation protein		HXT	−	LTA glycosylation [107]
**Adhesion**					
EF-P	Elongation factor EF-P	*L. acidophilus*	RSV [109]	+	Adhesion [112]
PK	Pyruvate kinase		RSV 109	+	Adhesion [113]
EF-Tu	Elongation factor EF-Tu		RSV [109]	+	Adhesion [114]
EF-P	Elongation factor EF-P		Caffeic acid [109]	−	Adhesion [112]
PYK	Pyruvate kinase		Caffeic acid [109]	−	Adhesion [113]
EF-Tu	Elongation factor EF-Tu		Caffeic acid [109]	−	Adhesion [114]
*lp_0728*	*groEL*: chaperonin *groEL*	*L. plantarum*	*p*-CA, HXT, OLE	+	Adhesion [117]
EF-GreA	Elongation factor EF-GreA	*L. plantarum*	TA [37]	+	Adhesion [110]
LuxS	Autoinducer production protein LuxS		TA [37]	−	Adhesion [111]
*lp_1229*	*msa*: mannose-specific adhesin	*L. plantarum*	RSV [27]	+	Mannose-specific adhesion [119]
*lp_1261*	*oppA*: oligopeptide ABC transporter substrate binding protein		*p*-CA, OLE	+	Adhesion [115]
*lp_0129*	*hsp1*: small heat-shock protein 1		*p*-CA	+	Adhesion [118]
*lp_3352*	*hsp3*: small heat-shock protein 3		*p*-CA	+	Adhesion [118]
**Immnunomodulation**					
*lp_3581a*	*lamD*: auto-inducing peptide (AIP) precursor	*L. plantarum*	OLE	−	Immunomodulatory capacity [107]
*lp_3581*	*lamC*: two-component system, LytTR family		OLE	−	Immunomodulatory capacity [107]
*lp_0422*	*plnE*: bacteriocin precursor peptide		OLE	−	Immunomodulatory capacity [107]
*lp_0421*	*plnF*: bacteriocin precursor peptide		OLE		Immunomodulatory capacity [107]
*lp_0419*	*plnI*: bacteriocin immunity protein		OLE		Immunomodulatory capacity [107]
*lp_0423*	*plnG*: bacteriocin ABC transporter, ATP-binding and permease protein		OLE	−	Immunomodulatory capacity [107]
*lp_0424*	*plnH*: bacteriocin ABC transporter, accessory factor		OLE	−	Immunomodulatory capacity [107]
*lp_0428*	*plnV*: membrane protein		OLE	−	Immunomodulatory capacity [107]
*lp_2647*	*pts19A*: PTS system,*N*-acetylglucosamine/galactosamine-specific EIIA component		OLE	−	Immunomodulatory capacity [108]
*lp_2650*	*pts19B*: PTS system, *N*-acetylglucosamine-specific EIIB component		OLE	−	Immunomodulatory capacity [108]
*lp_2460*	prophage P2b protein 21		OLE	−	Immunomodulatory capacity [108]

^a^ Reference describing the regulation of the molecular actor (gene or protein) by the plant phenolic compound. *p*-CA (*p*-coumaric acid), RSV (resveratrol), OLE (oleuropein), HXT (hydroxytyrosol), TA (tannic acid); ^b^ reference describing the phenotype associated with the molecular actor (gene or protein); +, upregulation; −, downregulation.

**Table 5:** Structural formulas of plant phenolic compounds and techniques used to study the molecular responses of *Lactobacillus* spp. to these compounds.

Phenolic Compound	Technique	Species	References
**Hydroxycinnamic acids**			
***p-*** **coumaric acid** 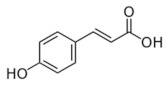	Transcriptomics Proteomics	*L. plantarum* *L.casei*	[25] [24]
**Ferulic acid** 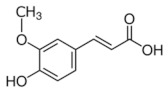	Transcriptomics Proteomics	*L. brevis* *L. acidophilus*	[23] [109]
**Caffeic acid** 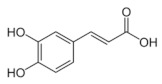	Proteomics	*L. acidophilus*	[109]
**Hydroxybenzoic acids**			
**Gallic acid** 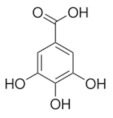	Transcriptomics	*L. plantarum*	[26]
**Stilbenoids**			
**Resveratrol** 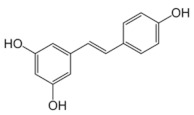	Transcriptomics Proteomics	*L. plantarum* *L. acidophilus*	[27] [109]
**Phenylethanoid**			
**Hydroxytyrosol** 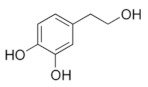	Transcriptomics	*L. plantarum*	[29]
**Flavonoids**			
**Rutin** 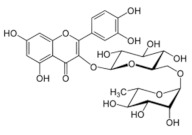	Proteomics	*L. acidophilus*	[45]
**Secoiridoids**			
**Oleuropein** 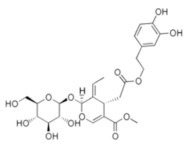	Transcriptomics	*L. plantarum*	[28]
**Tannins**			
**Tannic acid** 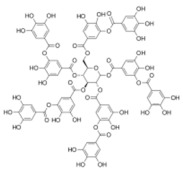	Proteomics Differential gene expression	*L. plantarum* *L. plantarum*	[37,43,44] [64]

## Data Availability

The data presented in this study are available in review.
